# Associations Between Patients' Primary Spoken Language and Perioperative Outcomes After Hip Fracture

**DOI:** 10.5435/JAAOSGlobal-D-25-00110

**Published:** 2025-07-02

**Authors:** Avanish Yendluri, Mateo Restrepo Mejia, Brocha Z. Stern, Charles Laurore, Rodnell Busigo Torres, Steven Yacovelli, Carolina Stocchi, Jashvant Poeran, Jeremy D. Podolnick, David A. Forsh

**Affiliations:** From the Leni and Peter W. May Department of Orthopedic Surgery, Icahn School of Medicine at Mount Sinai (Mr. Yendluri, Mr. Restrepo Mejia, Dr. Stern, Mr. Laurore, Busigo Torres, Dr. Yacovelli, Stocchi, Dr. Podolnick, Dr. Forsh); the Department of Population Health Science and Policy, Institute for Health Care Delivery Science, Icahn School of Medicine at Mount Sinai, New York, NY (Dr. Stern); and the Department of Anesthesiology, Critical Care and Pain Management, Hospital for Special Surgery (Dr. Poeran), New York, NY.

## Abstract

**Introduction:**

Recently, the role of a patient's primary spoken language (PSL) has emerged as a potential contributor to perioperative outcomes. This study aimed to identify the association between a patient's PSL and hip fracture perioperative outcomes, including hospital length of stay (LOS), in-hospital mortality, nonhome discharge, 30-day emergency department visits, 90-day readmissions, and 90-day complications.

**Methods:**

An institutional review board–approved retrospective cohort study was performed using institutional data. Two cohorts were created for patients who underwent either open reduction and internal fixation (ORIF) or arthroplasty (total or hemiarthroplasty) for a hip fracture in 2016 to 2023 in a multihospital academic health system. Within each cohort, patients with non-English and English PSL were matched 1:3 based on age, sex, insurance, American Society of Anesthesiologists Classification, dementia, and obesity. Generalized linear models measured associations between PSL and outcomes, adjusting for Charlson Comorbidity Index; adjusted odds ratios are reported for binary outcomes and adjusted mean differences are reported for continuous outcomes.

**Results:**

The matched cohorts included 729 patients undergoing ORIF and 473 undergoing arthroplasty. In multivariable analyses in the ORIF cohort, non-English PSL (versus English) was associated with a markedly longer LOS by an average of 0.95 days (95% confidence interval [CI], 0.28 to 1.62, *P* = 0.01) and decreased likelihood of discharge to a nonhome setting (odds ratio = 0.62, 95% confidence interval, 0.39 to 0.98, *P* = 0.04). For arthroplasty, non-English PSL (versus English) was markedly associated with shorter LOS by an average of 1.04 days (95% CI, −1.99 to −0.09, *P* = 0.03). No notable associations were identified between PSL and the other outcomes.

**Conclusion:**

These findings suggest language-based differences in perioperative outcomes for surgical management of hip fracture. Further research is needed to identify the mechanisms of these associations and evaluate the clinical significance on long-term outcomes.

Hip fractures are a growing concern, with considerable morbidity and mortality particularly among older patients^1-5^. Although mortality rates after hip fracture have been steadily declining, a 22% mortality rate still exists within 1 year of surgical intervention for patients 65 years and older.^2^ Prior literature has highlighted the role of disparities based on race, ethnicity, and socioeconomic status in the surgical management of patients with hip fractures.^6-8^ Recently, the role of a patient's primary spoken language (PSL) has emerged as a potential contributor to longer length of stay (LOS), greater hospitalization costs, and a decreased likelihood of being discharged home.^9-15^

There currently remains a knowledge gap regarding the role of a patient's PSL on surgical care among patients with a hip fracture. Although online and telephone interpreters are increasingly prevalent in emergency departments (EDs) and hospital settings to enhance access to care for patients with non-English PSL, it remains uncertain whether they mitigate disparities in perioperative outcomes. Given the health care costs, effect on quality of life, and mortality rates among elderly patients with hip fractures,^16^ it is important to elucidate the role that PSL has on the outcomes of patients undergoing surgical care for a hip fracture.

The purpose of this study was to examine the relationship between a patient's PSL and pertinent patient outcomes after surgical management of hip fractures at a major urban academic institution. We hypothesized that patients with non-English PSL undergoing open reduction and internal fixation (ORIF) or arthroplasty would experience longer hospital LOS, a lower likelihood of being discharged home, higher readmission rates, and higher complication rates compared with those with English PSL.

## Methods

### Design and Cohort

**Figure 1:**
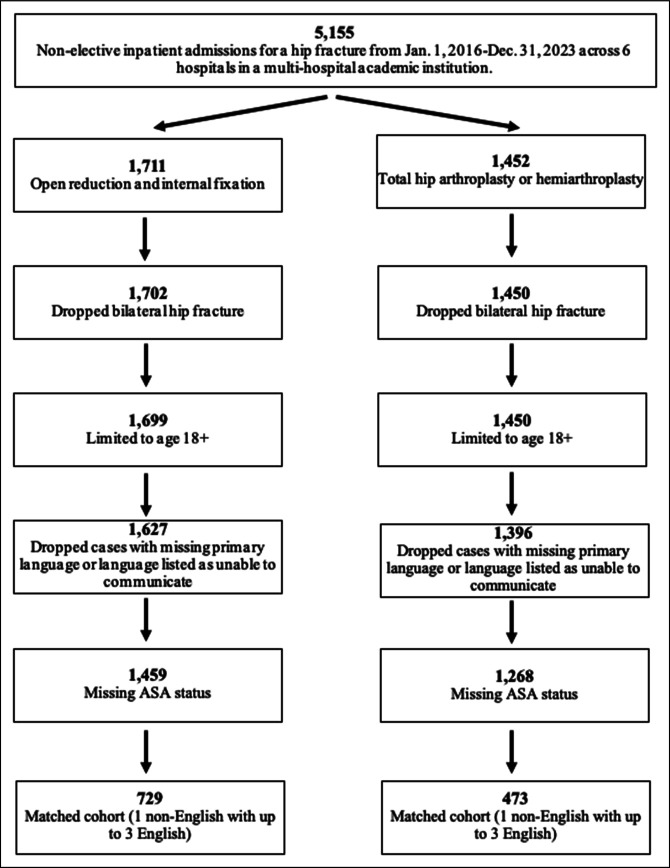
Flowchart showing cohort creation.

This was a retrospective cohort study using institutional data from a multihospital academic health system in the Northeast United States. Institutional review board approval was obtained (STUDY-21-00918). Patients were eligible if they were aged 18+ years and underwent a nonelective surgery for an intracapsular (ICD-10 diagnosis code S720**A) or extracapsular (ICD-10 diagnosis code S721**A, S722**A) proximal femur fracture with an inpatient admission between January 1, 2016, and December 31, 2023, at one of six hospitals in the system (Figure 1). The patients were separated into two cohorts by procedure type: (1) ORIF and (2) arthroplasty (hip hemiarthroplasty and total hip arthroplasty; see procedure codes in Appendix Table A1, http://links.lww.com/JG9/A417). Patients with bilateral hip fractures or with missing American Society of Anesthesiologists Classification (ASA) were excluded. In addition, patients missing PSL information or with language listed as unable to communicate were excluded.

### Variables of Interest

The patient's PSL was extracted from the medical record and categorized as non-English versus English. Although we did not have information on whether interpretation services were used for the specific encounter, our health system has standard procedures in place for those who speak a language other than English. Specifically, a third-party company is used for phone and video interpretation during all in-hospital interactions with non–English-speaking patients (e.g., consenting, daily rounds, and discharge). Phone services support more than 200 languages, and video is available in 35 languages. Discharge instructions can also be printed in more than 35 languages. These services are used by attendings, residents, physician assistants, nursing staff, and other allied health care professionals. Providers fluent in a patient's primary language may opt out if they have been certified to use that language in a medical setting.

The outcomes of interest included hospital LOS (in days), in-hospital mortality, nonhome discharge (i.e., discharge to inpatient acute or postacute care), 30-day ED visit, 90-day readmission, and 90-day complications (venous thromboembolism [VTE], infection, or bleeding). All outcomes except LOS and in-hospital mortality were evaluated in those discharged alive. Demographic and clinical variables extracted from the medical record were age (in years), sex (male or female), race/ethnicity (White, Asian, Black, Hispanic, other), insurance status (fee-for-service Medicare, Medicare Advantage, commercial, other), ASA physical status classification (1 or 2, 3, 4), Charlson Comorbidity Index (CCI) scores, dementia status (yes/no), tobacco use status (yes/no), and obesity status (yes/no). In addition, for the arthroplasty cohort, the procedure type was classified as total hip arthroplasty or hip hemiarthroplasty.

### Data Analysis

Statistical analyses were performed using SAS v9.4 (SAS Institute) and were done separately for the ORIF and arthroplasty cohorts. To minimize confounding, a greedy matching without replacement algorithm was used to match patients with non-English versus English PSL based on demographic and clinical characteristics. Specifically, one patient with a non-English PSL was matched with up to three patients with an English PSL based on the following variables: age ± 1 year, sex, insurance status (fee-for-service Medicare, Medicare Advantage, commercial/other) ASA status (1/2, 3, 4), dementia status (yes/no), and obesity status (yes/no). Descriptive statistics (frequencies and percentages or medians and interquartile ranges [IQRs]) were calculated for demographic and clinical variables within each matched cohort. Between-group differences were assessed for non-English versus English PSL using the chi-square test or Fisher exact test for categorical variables and the Mann-Whitney *U*-test for continuous variables.

Generalized linear models were used in the matched cohorts to measure associations between PSL and outcomes of interest, further adjusting for CCI (as a continuous score). The CCI score was not included in the matching algorithm itself for parsimony. Although we matched on dementia, we also included it as a covariate in the model to ensure that it was fully adjusted for, given its clinical importance in outcomes such as discharge destination. When model fit supported its inclusion, hospital was included as a random intercept to account for clustering of patients within sites. For the continuous outcome of hospital LOS, a gamma distribution and log link were used, and model coefficients were converted to adjusted means and 95% confidence intervals (CIs) using the least squares means function in SAS. For categorical outcomes, a binary distribution and logit link were used, and adjusted odds ratios (ORs) and 95% CIs are reported. When a statistically significant association was identified between PSL and an outcome in the multivariable models, further analysis decomposed the language exposure into Spanish versus English and other non-English versus English. The primary models were not adjusted for race/ethnicity because PSL may be a mechanism for race-based differences, and adjusting for these related variables in the same model may mask associations of interest. However, the models were further adjusted for race/ethnicity (collapsed as White/non-White) in sensitivity analyses to confirm the pattern of findings.

## Results

### ORIF Cohort

Before matching, there were 1,459 patients in the ORIF cohort (17.0% with a non-English PSL). After matching, the cohort included 729 patients, including 213 patients with a non-English PSL and 516 patients with an English PSL. The most common non-English languages were Spanish (n = 110), Russian (n = 32), Cantonese (n = 12), and Greek (n = 12). Overall, the median age was 86 years (IQR 80-90 years), 62.1% of patients were White, and 70.5% of patients were female (Table 1). Median CCI was 1 (IQR 0-2), and 32.5% of patients had dementia. English and non-English PSL patients who underwent ORIF differed by race (*P* < 0.001) and CCI (*P* = 0.01; Table 1).

**Table 1:** Demographic and Clinical Characteristics for Patients Undergoing Hip Fracture Surgery Stratified by Procedure Type and Primary Spoken Language

Variable	Category	ORIF (Matched Cohort)			Arthroplasty (Matched Cohort)		
		Non-English (n = 213), n (%)	English (n = 516), n (%)	*P* ^a^	Non-English (n = 140), n (%)	English (n = 333), n (%)	*P* ^a^
Age (median, IQR)		86 (80-90)	86 (80-90)	0.82	84 (77-89.5)	84 (77-90)	0.89
Sex	Male	67 (31.5)	148 (28.7)	0.45	39 (27.9)	79 (23.7)	0.34
	Female	146 (68.5)	368 (71.3)		101 (72.1)	254 (76.3)	
Race^b^	White	72 (35.3)	356 (73.4)	<0.001	37 (27.6)	210 (67.1)	<0.001
	Asian	28 (13.7)	10 (2.1)		26 (19.4)	12 (3.8)	
	Black	1 (0.5)	42 (8.7)		1 (0.8)	36 (11.5)	
	Hispanic	66 (32.4)	38 (7.8)		41 (30.6)	21 (6.7)	
	Other	37 (18.1)	39 (8.0)		29 (21.6)	34 (10.9)	
Insurance status	Fee-for-service Medicare	90 (42.3)	250 (48.5)	0.17	52 (37.1)	142 (42.6)	0.47
	Medicare advantage	104 (48.8)	221 (42.8)		65 (46.4)	145 (43.5)	
	Commercial	3 (1.4)	16 (3.1)		7 (5.0)	20 (6.0)	
	Other	16 (7.5)	29 (5.6)		16 (11.4)	26 (7.8)	
ASA^b^ status	1 or 2	30 (14.1)	74 (14.3)	0.95	13 (9.3)	32 (9.6)	0.90
	3	137 (64.3)	336 (65.1)		98 (70.0)	238 (71.5)	
	4	46 (21.6)	106 (20.5)		29 (20.7)	63 (18.9)	
CCI group	0	65 (30.5)	199 (38.6)	0.01	51 (36.4)	131 (39.3)	0.08
	1	75 (35.2)	193 (37.4)		40 (28.6)	116 (34.8)	
	2	43 (20.2)	87 (16.9)		28 (20.0)	60 (18.0)	
	3+	30 (14.1)	37 (7.2)		21 (15.0)	26 (7.8)	
Dementia	Yes	73 (34.3)	164 (31.8)	0.51	41 (29.3)	93 (27.9)	0.77
Tobacco use	Yes	5 (2.4)	15 (2.9)	0.67	3(2.1)	15 (4.5)	0.22
Obesity	Yes	5 (2.4)	6 (1.2)	0.31	0 (0.0)	0 (0.0)	
Surgery	Total hip arthroplasty	—	—	—	116 (82.9)	280 (84.1)	0.74
	Hemiarthroplasty	—	—	—	24 (17.1)	53 (15.9)	

ASA = American Society of Anesthesiologists Classification, CCI = Charlson Comorbidity Index score, ORIF = open reduction and internal fixation

a Between-group differences assessed using the chi-square test or Fisher exact for categorical variables and Mann-Whitney *U*-test.

b Missing race/ethnicity of n = 40 for the ORIF cohort and n = 26 for the arthroplasty cohort.

In unadjusted analyses, there were notable differences in hospital LOS, with a longer median LOS for patients with non-English (7 days, IQR 5-10 days) versus English PSL (6 days, IQR 4.5 to 9 days; *P* = 0.01; Table 2). No other notable between-group unadjusted differences were found in outcomes (Table 2).

**Table 2:** Unadjusted Differences in Outcomes of Interest Stratified by Procedure Type and Primary Spoken Language

Variable	ORIF (Matched Cohort)			Arthroplasty (Matched Cohort)		
	Non-English (n = 213), n (%)	English (n = 516), n (%)	P^a^	Non-English (n = 140), n (%)	English (n = 333), n (%)	P^a^
Hospital length of stay (median, IQR)	7 (5-10)	6 (4.5-9)	0.01	7 (5-9)	7 (5-10)	0.25
In-hospital mortality (yes)	4 (1.9)	7 (1.4)	0.74	2 (1.4)	7 (2.1)	1.0
Nonhome discharge (yes)^b^	38 (18.4)	73 (14.5)	0.20	21 (15.6)	47 (14.6)	0.78
30-day emergency department visit (yes)	20 (9.6)	58 (11.4)	0.48	16 (11.6)	30 (9.2)	0.43
90-day readmission (yes)	45 (21.5)	110 (21.6)	0.98	26 (18.8)	52 (16.0)	0.45
90-day venous thromboembolism (yes)	2 (1.0)	5 (1.0)	1.0	3 (2.2)	5 (1.5)	0.7
90-day bleeding-related complication (yes)	0 (0)	0 (0)	N/A	0 (0)	0 (0)	N/A
90-day postoperative infection (yes)	0 (0)	0 (0)	N/A	0 (0)	0 (0)	N/A

ORIF = open reduction and internal fixation

a Between-group differences assessed using the chi-square test or Fisher exact for categorical variables and Mann-Whitney *U*-test for continuous variables.

b Unable to classify discharge disposition for n = 8 for ORIF cohort and n = 6 for arthroplasty cohort.

In multivariable analyses, non-English PSL patients who underwent ORIF had a markedly longer LOS by an average of 0.95 days (95% CI, 0.28 to 1.62, *P* = 0.01; Table 3). Specifically, patients with other non-English versus English PSL had a markedly longer LOS by an average of 1.7 days (95% CI, 0.70 to 2.62, *P* = 0.001), but Spanish versus English PSL had no notable association with LOS (mean difference of 0.29 days, 95% CI, −0.52 to 1.10, *P* = 0.49). Furthermore, patients with non-English PSL who underwent ORIF were markedly less likely to be discharged to a nonhome setting compared with English PSL patients (OR = 0.62, 95% CI, 0.39 to 0.98, *P* = 0.04, Table 3). Specifically, patients with non-English PSL other than Spanish were markedly less likely to be discharged to a nonhome setting than those with English PSL (OR = 0.53, 95% CI, 0.28 to 0.99, *P* = 0.045), but no difference was found for Spanish versus English (OR = 0.69, 95% CI, 0.39 to 1.22, *P* = 0.20). No notable associations were identified between PSL and in-hospital mortality, 30-day ED visit, 90-day readmission, and 90-day VTE for patients who underwent ORIF (Table 3). Overall, findings were similar when further adjusting for race/ethnicity in the sensitivity analyses. However, the association between PSL and nonhome discharge was no longer notable (*P* = 0.06; Appendix Table A2, http://links.lww.com/JG9/A418).

**Table 3:** Associations Between Non-English and English primary spoken language and Hip Fracture Surgery Outcomes

Outcome Variable	ORIF (Matched Cohort)			Arthroplasty (Matched Cohort)		
	Adjusted Mean Difference	95% CI	*P*	Adjusted Mean Difference	95% CI	*P*
Hospital length of stay	+0.95	0.28-1.62	0.01	−1.04	−1.99-−0.09	0.03

Outcome Variable	ORIF (matched cohort)			Arthroplasty (matched cohort)		
	Adjusted OR	95% CI	*P*	Adjusted OR	95% CI	*P*
In-hospital mortality	1.23	0.35-4.36	0.75	0.585	0.12-2.95	0.52
Nonhome discharge	0.62	0.39-0.98	0.04	0.89	0.50-1.56	0.68
30-day emergency department visit	0.69	0.40-1.21	0.19	1.17	0.61-2.27	0.64
90-day readmission	0.82	0.54-1.24	0.34	1.13	0.67-1.93	0.65
90-day venous thromboembolism	1.02	0.17-5.08	0.93	1.36	0.31-5.91	0.68

ORIF = open reduction and internal fixation

All point estimates are for non-English versus the reference group of English primary spoken language and are adjusted for Charlson Comorbidity Index score. All outcomes except hospital length of stay and in-hospital mortality are for patients discharged alive.

### Arthroplasty Cohort

Before matching, there were 1,268 patients in the arthroplasty cohort (12.9% with a non-English PSL). After matching, the cohort included 473 patients, including 140 patients with a non-English PSL and 333 patients with an English PSL. The most common non-English languages were Spanish (n = 68), Cantonese (n = 14), and Russian (n = 14). Overall, the median age was 84 years (IQR 77-90 years), 55.3% of patients were White, and 75.1% of patients were female (Table 1). Median CCI was 1 (IQR 0-2), and 28.3% of patients had dementia. In addition, 83.7% underwent total hip arthroplasty and 16.3% underwent hip hemiarthroplasty. The only between-group difference in the matched cohort was race (*P* < 0.001; Table 1).

In unadjusted analyses, no notable between-group differences were found in outcomes for English and non-English PSL patients who underwent arthroplasty (Table 2).

However, in multivariable analyses, non-English PSL patients who underwent arthroplasty had a markedly shorter LOS by an average of 1.04 days (95% CI, −1.99 to −0.09, *P* = 0.03; Table 3) compared with English PSL patients. Specifically, patients with Spanish PSL had a markedly shorter LOS by an average of 1.5 days (95% CI, −2.71 to −0.38, *P* = 0.01), but no difference was found for other non-English versus English (mean difference of −0.55 days, 95% CI, −1.81 to 0.72, *P* = 0.40). No notable associations were identified between PSL and in-hospital mortality, nonhome discharge, 30-day ED visit, 90-day readmission, and 90-day VTE for patients who underwent arthroplasty (Table 3). Findings were similar when further adjusting for race/ethnicity in the sensitivity analyses (Appendix Table A2, http://links.lww.com/JG9/A418).

## Discussion

The purpose of the present study was to evaluate the association between patients' PSL and perioperative outcomes for patients who underwent either ORIF or arthroplasty for a hip fracture. In the matched ORIF cohort that accounted for confounders, we identified associations between non-English versus English PSL and increased hospital LOS and decreased nonhome discharge. In the matched arthroplasty cohort, we identified an association between non-English versus English PSL and decreased hospital LOS. No notable associations were identified between PSL and in-hospital mortality, 30-day ED visit, 90-day readmission, and 90-day complications for either cohort.

A 2024 systematic review highlighted the effect of limited English proficiency on clinical care processes, patient engagement, and outcomes in orthopaedic surgery, particularly in elective lower extremity joint replacement.^17^ Importantly, however, research evaluating language-based disparities for patients in the nonelective orthopaedic trauma care setting remains limited.

Specifically for hospital LOS, there are mixed findings on the effect of language barriers in orthopaedic surgery. Longer LOS has been identified in patients with limited English proficiency undergoing total joint or shoulder arthroplasty,^11,13^ whereas other studies found no notable differences in LOS between English-speaking and non-English-speaking patients undergoing total knee arthroplasty or hip fracture.^18,19^ These conflicting findings highlight the potential importance of considering language diversity within non–English-speaking populations. Our research further supports this by demonstrating that only patients from non–English-speaking backgrounds, other than Spanish, experienced longer LOS when compared with English speakers in the ORIF cohort. Interestingly, we found that Spanish-speaking patients undergoing arthroplasty had a markedly shorter LOS compared with their English-speaking counterparts. Spanish-speaking patients in this cohort may benefit from strong family or community support systems that help facilitate a quicker recovery and discharge process. Another possibility is that hospital systems serving larger Spanish-speaking populations may have developed more streamlined, culturally competent care pathways for these patients, resulting in more efficient care and quicker discharges. This is in line with a study published by Feeney et al,^20^ which found that Spanish-speaking patients experienced a markedly shorter LOS after emergency general surgery. These findings underscore the necessity of segmenting study results by specific language groups for a more nuanced understanding of how language influences orthopaedic care outcomes. Importantly, a 2023 study by Kunze et al^11^ emphasized that language barriers or inconsistent interpreter use in time-sensitive situations may contribute to inadequate communication of key information between hospital staff and patients and thus affect readiness for discharge. The resultant differences in LOS and hospitalization may also affect healthcare costs with considerable implications for healthcare administrators.^13^

For discharge disposition, several studies have identified increased nonhome discharge after elective orthopaedic surgery for patients with limited English proficiency.^11,13,14,19^ By contrast, our study showed no association between PSL and discharge disposition for patients undergoing arthroplasty and identified that non–English-speaking patients who underwent ORIF were less likely to be discharged to places other than their homes and that this was particularly true for patients with a non-English PSL other than Spanish. However, this estimate should be interpreted with caution because it was no longer notable after adjusting for race/ethnicity. The discrepancies compared with past findings may be potentially related to differences in elective versus trauma contexts. In the elective context, patients with English proficiency may have a greater advantage over peers with limited English proficiency through increased access to preoperative educational resources that could facilitate discharge home. By contrast, in the nonelective context, the finding of decreased discharge to specialized postacute facilities may be at least attributable to difficulties in communicating potential benefits of nonhome discharge options such as inpatient rehabilitation to patients whose primary language is not English. This aligns with a study by Maurer et al,^21^ which highlighted the complexities in eligibility, approval, and physician-patient communication when discharging a patient to inpatient rehabilitation that may be hindered by language barriers.

For postoperative return to the hospital, including ED visits and readmissions, hospital readmissions, a study on total knee arthroplasty indicated that non–English-speaking patients were more likely to be readmitted within 90 days but not 30 days when compared with those with English PSL.^19^ Conversely, a study in Ontario, Canada, found no notable association between language and ED visits or readmissions (both 30 and 90 day) among patients who had sustained a hip fracture.^22^ Similarly, our findings revealed no notable correlations between language and 30-day ED visits or 90-day readmissions in both of our patient cohorts. Overall, the limited consensus across study results suggest that the outcomes may be influenced by the linguistic diversity of the patient population and the availability of language support programs within different healthcare facilities.

Regarding in-hospital mortality and postoperative complications, the existing research on the relationship with language barriers is limited. Castro et al identified that trauma patients who spoke Spanish and Chinese had a higher mortality rate compared with those who spoke English.^23^ Conversely, a systematic review by Woods et al,^24^ which focused on disparities among patients with limited English proficiency in hospital settings, found that 13 of 16 studies showed no link between mortality and the language spoken. Specific to hip fracture, a study found no association between non-English PSL and in-hospital mortality.^18^ For other complications, Nguyen et al^9^ found that patients who do not speak English and underwent total knee arthroplasty were at a higher risk of developing deep vein thrombosis and VTE within 30 days postoperatively compared with their English-speaking counterparts. However, a study in total shoulder arthroplasty found no notable differences in complications by language.^11^ In our study, we also observed no notable differences for in-hospital mortality or 90-day complications, including VTE, bleeding, and infection, in both the ORIF and arthroplasty cohorts based on PSL.

## Limitations

The study's findings are subject to several limitations inherent in retrospective cohort analyses, including the inherent challenge of controlling for all potentially confounding factors. However, we employed a robust matched cohort analysis separated by procedure type to mitigate this issue.

Our data set also has limited details of outcomes, such as reasons underlying readmission or ED visit, which could identify differences in mechanism of outcomes even if the rate of outcomes are similar. This study also spans the COVID-19 pandemic, which may have influenced complication rates or LOS of patients. In addition, although the study included data from a multihospital system, findings may not generalize beyond this single academic health system to other regions or healthcare settings with different patient demographics or language support services. Furthermore, the limited sample size for the non–English-speaking patients combined with the number of small events for some outcomes may have limited the ability to detect statistically significant associations due to the risk of type II errors. In addition, the exposure of PSL as defined in this study is a rough proxy for English language proficiency. We did not have information on degree of English proficiency and patient education or health literacy, which could have biased results. In addition, to facilitate matching, language was collapsed into non-English versus English for the primary analyses, which may mask important subgroup differences for less commonly spoken languages. The study also does not account for the possible influence of interpreter services' availability or quality, nor does it consider the role of non-English language proficiency or cultural competence among members of the care team, which could affect patient care and satisfaction. For example, information is not available on whether education about the diagnosis, procedure, or postoperative care was provided directly by a bilingual healthcare professional, through a qualified interpreter, or through a companion. The education level, level of English proficiency, or bilingualism of patients or the varying levels of bilingualism among members of the patient's care team was also not available in our data set. Thus, our analysis was limited to using PSL as a proxy. Finally, we acknowledge that our data set lacked important surgical details (e.g., quality of reduction, cement usage, surgical duration, estimated blood loss) and also did not capture information on patients' support networks or socioeconomic status. Both factors may markedly influence perioperative outcomes but were unavailable for inclusion in this analysis. These limitations suggest the need for future research to adopt a more nuanced approach in evaluating the effects of language barriers on healthcare outcomes, incorporating measures of patient English proficiency, the quality of interpreter services, and cultural competency factors. Future research should also take into account patient-reported outcome measures to inform clinical decision making, while taking into account varying levels of English proficiency.^25^ Finally, future research should focus on developing and evaluating solutions to provide equitable health care to non-English speaking patients (i.e. multilingual chatbot to engage patients with limited English proficiency^26^). This understanding could enable institutions to offer enhanced resources for non–English-speaking patients, with the aim of bridging the disparity in an evidence-based manner.

## Conclusion

This study revealed several notable associations between patients' PSL and perioperative outcomes after hip fracture surgeries. For patients undergoing ORIF, non-English PSL, particularly languages other than Spanish, was associated with a longer hospital LOS and decreased likelihood of nonhome discharge. Conversely, Spanish-speaking patients undergoing arthroplasty experienced a shorter LOS. No marked differences were found in discharge dispositions among patients undergoing arthroplasty, nor were there notable associations with in-hospital mortality, 30-day ED visits, 90-day readmissions, or 90-day complications across both surgical cohorts. These findings affirm the current literature that suggests the effect of language barriers on some perioperative outcomes, even in nonelective settings. Further research is needed to evaluate the clinical significance of these findings on long-term outcomes and ways to address these barriers and bridge gaps in care for patients with limited English proficiency.
